# Reducing hand radiation during renal access for percutaneous nephrolithotomy: a comparison of radiation reduction techniques

**DOI:** 10.1007/s00240-023-01510-x

**Published:** 2024-01-13

**Authors:** Ricky Chen, Eun Hye Joo, Catalina Baas, John Hartman, Akin S. Amasyali, Kanha Shete, Joshua D. Belle, Cayde Ritchie, Elizabeth A. Baldwin, Zhamshid Okhunov, Ala’a Farkouh, D. Duane Baldwin

**Affiliations:** Department of Urology, Loma Linda University Health, Room A560, 11234 Anderson Street, Loma Linda, CA 92354 USA

**Keywords:** Fluoroscopy, Percutaneous nephrolithotomy, Radiation protection, Protective gloves, Radiation exposure

## Abstract

Percutaneous nephrolithotomy confers the highest radiation to the urologist’s hands compared to other urologic procedures. This study compares radiation exposure to the surgeon’s hand and patient’s body when utilizing three different techniques for needle insertion during renal access. Simulated percutaneous renal access was performed using a cadaveric patient and separate cadaveric forearm representing the surgeon’s hand. Three different needle-holding techniques were compared: conventional glove (control), a radiation-attenuating glove, and a novel needle holder. Five 300-s fluoroscopy trials were performed per treatment arm. The primary outcome was radiation dose (mSv) to the surgeon’s hand. The secondary outcome was radiation dose to the patient. One-way ANOVA and Tukey’s B post-hoc tests were performed with *p* < 0.05 considered significant. Compared to the control (3.92 mSv), both the radiation-attenuating glove (2.48 mSv) and the needle holder (1.37 mSv) reduced hand radiation exposure (*p* < 0.001). The needle holder reduced hand radiation compared to the radiation-attenuating glove (*p* < 0.001). The radiation-attenuating glove resulted in greater radiation produced by the C-arm compared to the needle holder (83.49 vs 69.22 mGy; *p* = 0.019). Patient radiation exposure was significantly higher with the radiation-attenuating glove compared to the needle holder (8.43 vs 7.03 mSv; *p* = 0.027). Though radiation-attenuating gloves decreased hand radiation dose by 37%, this came at the price of a 3% increase in patient exposure. In contrast, the needle holder reduced exposure to both the surgeon’s hand by 65% and the patient by 14%. Thus, a well-designed low-density needle holder could optimize radiation safety for both surgeon and patient.

## Introduction

Various technical and procedural modifications have been implemented intraoperatively to reduce fluoroscopy exposure to both urologists and patients, including proper shielding, low power fluoroscopy settings, and conservative use of fluoroscopy [1]. Although these measures significantly reduce radiation exposure to the operating room staff and patient, the surgeon’s hand remains susceptible to radiation exposure using these methods. This is concerning as ionizing radiation is a risk factor for malignancy and many other adverse effects [2, 3]. Surgeons who frequently work within the direct radiation beam may experience skin and nail pigment abnormalities, joint pain, and osteoarthritic changes [4–6]. Furthermore, the full understanding of the health effects of radiation to the hand are currently limited in existing literature [7, 8].

Compared to shock wave lithotripsy (SWL) and ureteroscopy, percutaneous nephrolithotomy (PCNL) confers the highest radiation to both the surgeon and patient [9–11]. Radiation exposure to the surgeon includes both scatter and direct radiation with direct exposure being exponentially greater. The most common scenario when surgeons experience direct radiation exposure is while holding the needle during percutaneous access [9, 12]. This becomes even more important as the indications for PCNL expand and as a greater number of urologists obtain their own access [13].

To mitigate this exposure, radiation-attenuating gloves and needle holders have been proposed as potential protective measures by reducing penetrating radiation and enabling removal of the hand from the direct radiation beam. However, their effectiveness during percutaneous renal access for PCNL has not been investigated. The aim of this study was to evaluate the effectiveness of a radiation-attenuating glove compared to a novel needle holder in reducing radiation exposure to the surgeon's hand.

## Methods

### Study design and set up

**Fig. 1:**
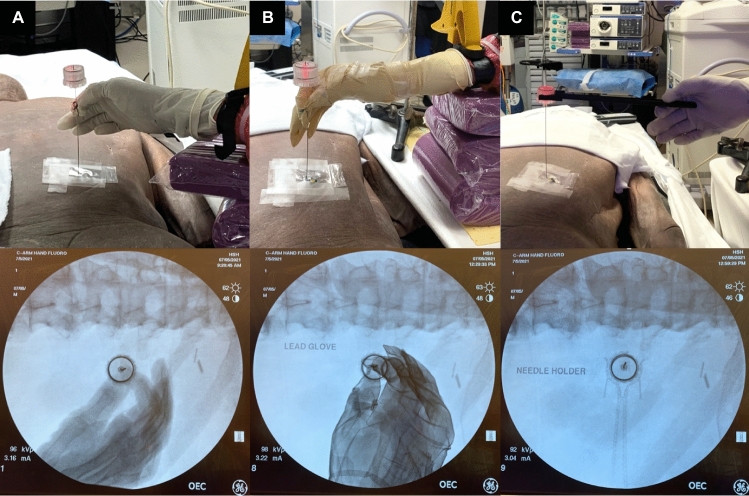
Cadaver hand representing a surgeon’s hand positioning and respective fluoroscopic images obtained during simulated percutaneous renal access on a cadaver patient model using **A** a surgical glove, **B** radiation-attenuating glove, and **C** needle holder

After approval from the Loma Linda University’s Department of Pathology and Human Anatomy, and in compliance with institutional policies for use of anatomical specimens in research, a simulated percutaneous renal access for PCNL was performed. A male cadaver (body mass index 36.1) was positioned prone and draped in a manner typical for PCNL. A separate cadaveric right upper extremity, representing the surgeon's hand, was positioned to simulate percutaneous right renal access with an access needle (Fig. 1).

### Radiation dose measurements

**Fig. 2:**
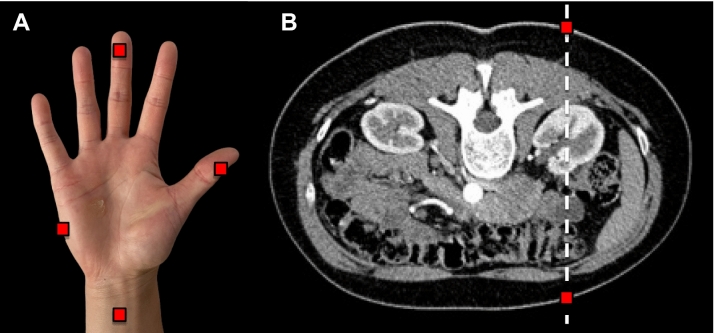
Optically stimulated luminescence dosimeter (OSLD) chips (red boxes) were fixed on **A** four locations of the surgeon hand model: the lateral distal phalanx of the first digit, ventral distal phalanx of the third digit, hypothenar eminence, and forearm 5 cm proximal to the anterior surface of the radiocarpal joint; **B** two OSLD chips were fixed on the patient model: the ventral and dorsal surface of the skin directly in line with the right kidney

Landauer nanoDot optically stimulated luminescence dosimeter (OSLD) chips (Glenwood, Illinois) were affixed to four locations on the surgeon's hand: the thumb, middle finger, hypothenar eminence, and forearm (Fig. 2). Additionally, two OSLD chips were placed on the patient: one on the ventral surface and one on the dorsal surface of the skin directly in line with the right kidney. The index finger was positioned 7.6 cm above the skin overlying the right kidney.

Fluoroscopy was performed using a GE OEC 9900 portable C-arm system (GE Medical system, Inc., Salt Lake City, UT) using the default automatic exposure control (AEC) for all trials. The AEC adjusts the milliampere-seconds (mAs) and peak kilovoltage (kVp) based on the target density to provide optimal image quality [14]. The C-arm was positioned over the right kidney with the X-ray source below the table and the image intensifier above the patient at a skin-to-source distance of 20 cm.

The study consisted of three groups. The first group served as the control with radiation exposure tested on the surgeon’s hand wearing conventional polyisoprene surgical gloves (Mölnlycke, Gothenburg, Sweden) directly holding the access needle. In the first experimental group, the renal access needle was held directly by the cadaveric arm using radiation-attenuating gloves (AliMed, Dedham, Massachusetts). The second experimental group employed a novel low radiodensity needle holder (Fig. 1), designed to facilitate PCNL access while ensuring that the surgeon’s hand is not directly in the line of the radiation beam.

Each treatment arm underwent five trials, with each trial having a fluoroscopy time of 300 s. This duration was chosen based on previous studies that reported the average fluoroscopy time during renal access for PCNL [15–18]. The OSLD chips used in the study were read with a microSTARii Dosimetry System (LANDAUER, Glenwood, Illinois). The absorbed dose measurements by OSLD chips were converted to equivalent doses in millisieverts (mSv) using the radiation weighting factor for fluoroscopy specified by the International Commission on Radiological Protection (ICRP) (*w*_R_ = 1) [19].

The radiation attenuating surgical gloves are constructed from a radiopaque proprietary material, which does not contain lead or latex. The interior is coated to allow easy donning. They are specifically designed to minimize the ionizing radiation penetrating the surgeon’s hand [20]. The needle holder employed in this study is 3D-printed with a 9-inch-long handle and a very low profile on fluoroscopy. The holder was specifically designed for use with a novel needle which includes a hub that securely interfaces with the needle holder for easy maneuverability and the potential for insertion using the handle. For consistency, this same needle was used in each of the three arms to eliminate it as a potential confounding variable.

The primary objective was to quantitatively measure and compare the radiation dose to the surgeon’s hand using the three different techniques for obtaining percutaneous renal access. The secondary outcomes were dose received by the patient, as well as the C-arm recorded cumulative radiation dose in mGy, current in mA, and voltage in kVp for each of the three arms.

### Statistical analysis

The data was statistically analyzed using one-way analysis of variance (ANOVA) and Tukey’s *B* post hoc test using SPSS version 24 (IBM, Armonk, NY). The significance threshold was set at *p* < 0.05.

## Results

**Fig. 3:**
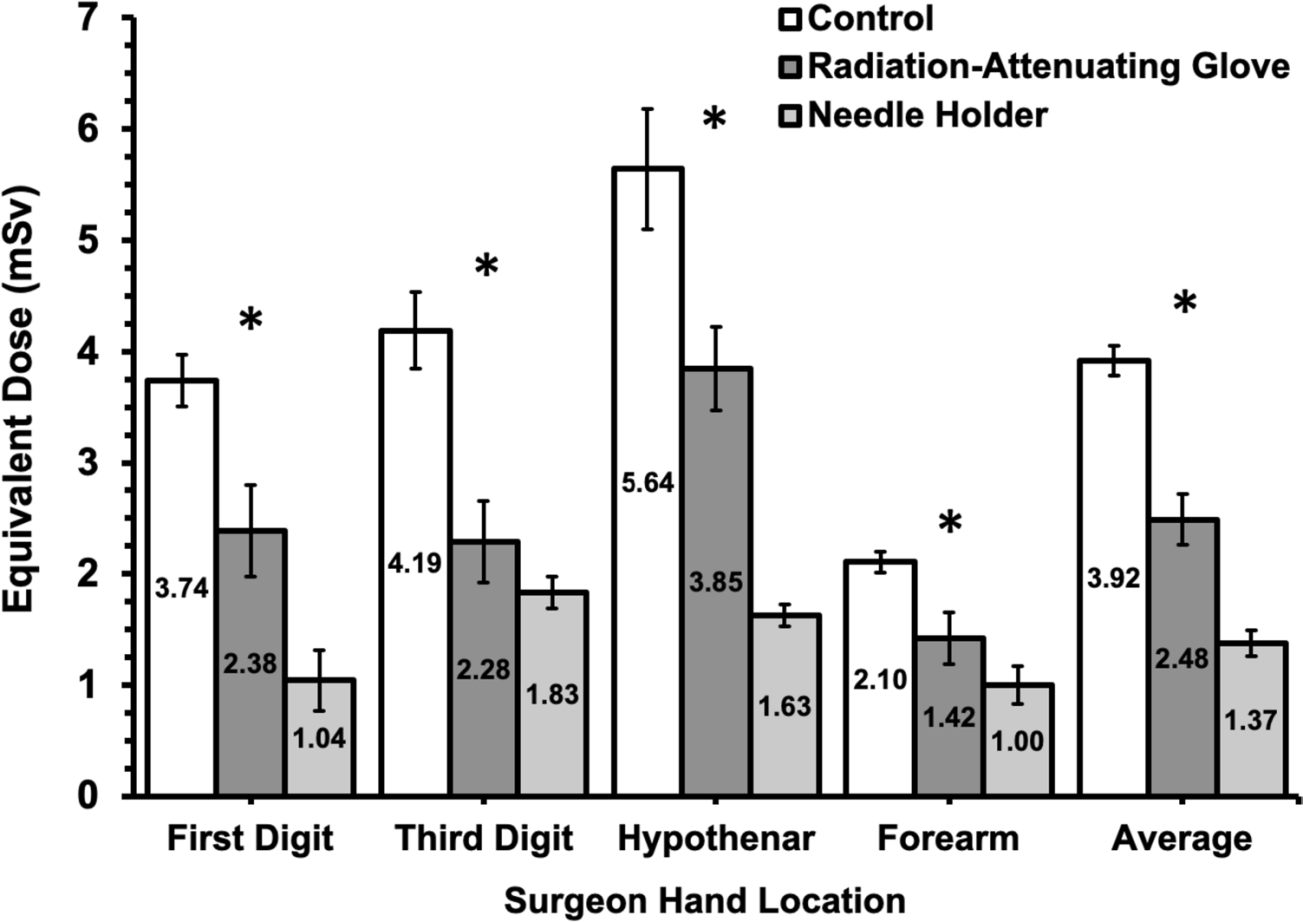
Mean equivalent dose of the first digit, third digit, hypothenar eminence, forearm, and overall average dose to the surgeon’s hand during simulated percutaneous renal access. Error bars represent one standard deviation. *p < 0.05 for all pairwise comparisons between arms except for the 3rd digit between the radiation-attenuating glove versus needle holder arms

During the 300 s of fluoroscopy with AEC settings, the surgeon’s hand in the control group received an average equivalent dose of 3.92 mSv (Table 1). Compared to the control, both the radiation-attenuating glove (2.48 mSv) and needle holder (1.37 mSv) reduced the average equivalent dose to the surgeon’s hand (*p* < 0.001; Table 2 and Fig. 3). The needle holder resulted in a significantly lower dose to the surgeon's hand than the radiation-attenuating glove (*p* < 0.001). At all locations on the surgeon's hand, both the radiation-attenuating glove and needle holder had lower doses compared to the control (*p* < 0.05 for all). The needle holder demonstrated a reduced dose that was significant at all locations except the middle finger (*p* = 0.082).

**Table 1:** Mean equivalent radiation dose and percent change versus control for the surgeon hand and patient between the control and radiation reduction techniques

	Control (*n* = 5)		Radiation-attenuating glove (*n* = 5)		Needle holder (*n* = 5)		*p* value
	Mean mSv (SD)	% Change vs control	Mean mSv (SD)	% Change vs control	Mean mSv (SD)	% Change vs control	
Surgeon hand							
Average	3.92 (0.13)	–	2.48 (0.23)	− 37	1.37 (0.11)	− 65	< 0.001*
Thumb	3.74 (0.23)	–	2.39 (0.41)	− 36	1.04 (0.27)	− 72	< 0.001*
Middle finger	4.19 (0.34)	–	2.29 (0.37)	− 45	1.83 (0.14)	− 56	< 0.001*
Hypothenar eminence	5.64 (0.54)	–	3.85 (0.38)	− 32	1.63 (0.10)	− 71	< 0.001*
Forearm	2.10 (0.10)	–	1.42 (0.24)	− 33	1.00 (0.17)	− 53	< 0.001*
Patient							
Dorsal surface	8.17 (0.83)	–	8.43 (0.90)	3	7.03 (0.31)	− 14	0.020*
Ventral surface	874.48 (44.92)	–	676.24 (303.801)	− 23	632.75 (269.67)	− 28	0.158

Bold indicates statsitically significant *p*-value

*n* number of patients, *mSv* millisievert, *SD* standard deviation

*p < 0.05

**Table 2:** Tukey post-hoc testing comparison of mean equivalent dose to the surgeon hand and patient model between the control and radiation reduction techniques

	Control	Radiation-attenuating glove	Needle holder
Surgeon hand average dose (mSv)*	3.92	2.48	1.37
Control	–	*p* < 0.001**	*p* < 0.001**
Radiation-attenuating glove		–	*p* < 0.001**
Needle Holder			–
Patient dorsal surface dose (mSv)	8.17	8.43	7.03
Control	–	*p* = 0.840	*p* = 0.073
Radiation-attenuating glove		–	*p* = 0.027**
Needle holder			–
Patient ventral surface dose (mSv)	874.48	676.24	632.75
Control	–	*p* = 0.407	*p* = 0.275
Radiation-attenuating glove		–	*p* = 0.954
Needle holder			–

Bold indicates statsitically significant *p*-value

*mSv* millisievert

*All other pairwise comparisons for surgeon hand dose were statistically significant except for third digit for radiation-attenuating glove vs needle holder

**p < 0.05

Within the control group, the hypothenar eminence received the highest dose (5.64 mSv), while the forearm received the lowest (2.10 mSv; *p* < 0.001; Fig. 3). The doses for the middle finger (4.19 mSv) and the thumb (3.74 mSv) were similar (*p* = 0.207). In the radiation-attenuating glove condition, only the hypothenar eminence received a significantly higher dose compared to the other locations (Table 1). In the needle holder condition, the middle finger received the highest dose compared to the first digit and forearm, but it was not significantly greater than the hypothenar eminence (Table 1).

**Fig. 4:**
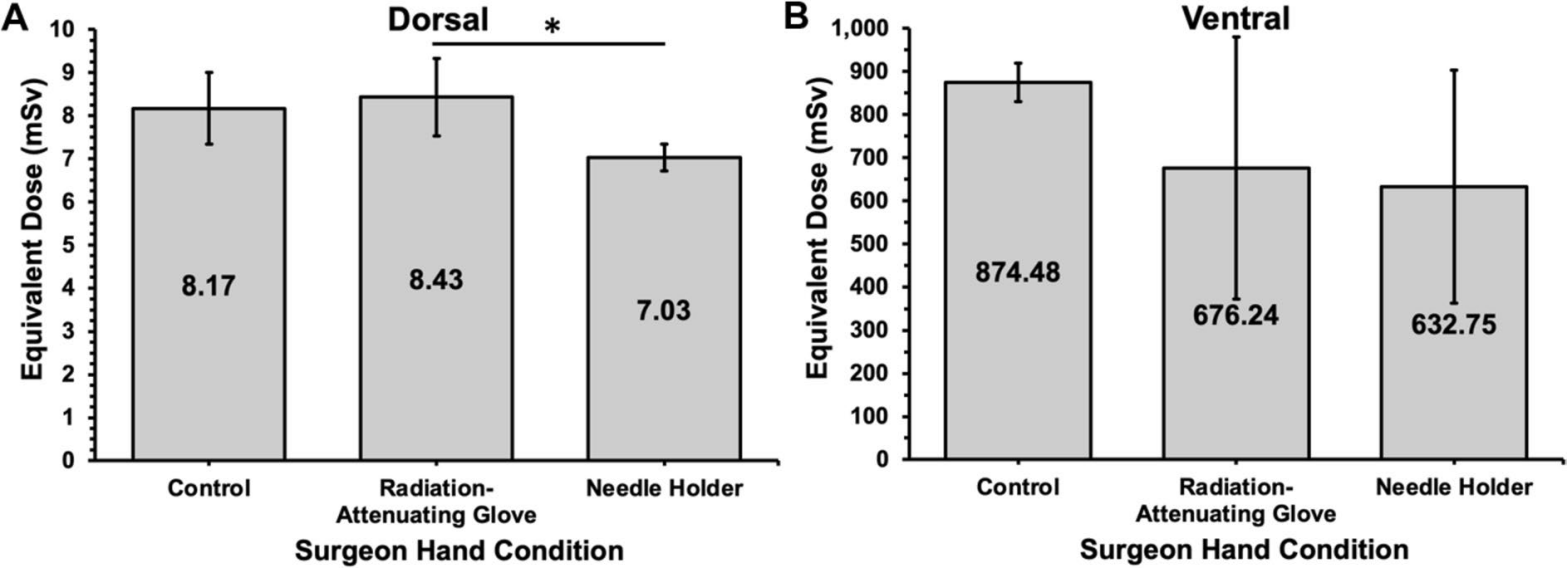
Mean equivalent dose of **A** the dorsal and **B** ventral surface of the patient with either the control, radiation-attenuating glove, or needle holder experimental arms during renal access for percutaneous nephrolithotomy. *p < 0.05

Compared to the control (8.17 mSv), the mean equivalent dose to the dorsal surface of the patient was greater when using a radiation-attenuating glove (8.43 mSv) and less when using the needle holder (7.03 mSv), though these differences were not significant (*p* > 0.05 for all; Table 1 and Fig. 4). However, the radiation-attenuating glove resulted in a significant increase in equivalent dose to the dorsal surface of the patient compared to the needle holder (8.43 vs 7.03 mSv; *p* = 0.027). No significant differences were found in the equivalent dose to the ventral surface of the patient between surgeon hand conditions (*p* > 0.05 for all). Overall, the ventral surface had a significantly higher mean equivalent dose than the dorsal surface of the patient for the control (874.48 vs 8.17 mSv), radiation-attenuating glove (676.24 vs 8.43 mSv), and needle holder (632.75 vs 7.03 mSv) conditions (*p* < 0.001 for all).

**Fig. 5:**
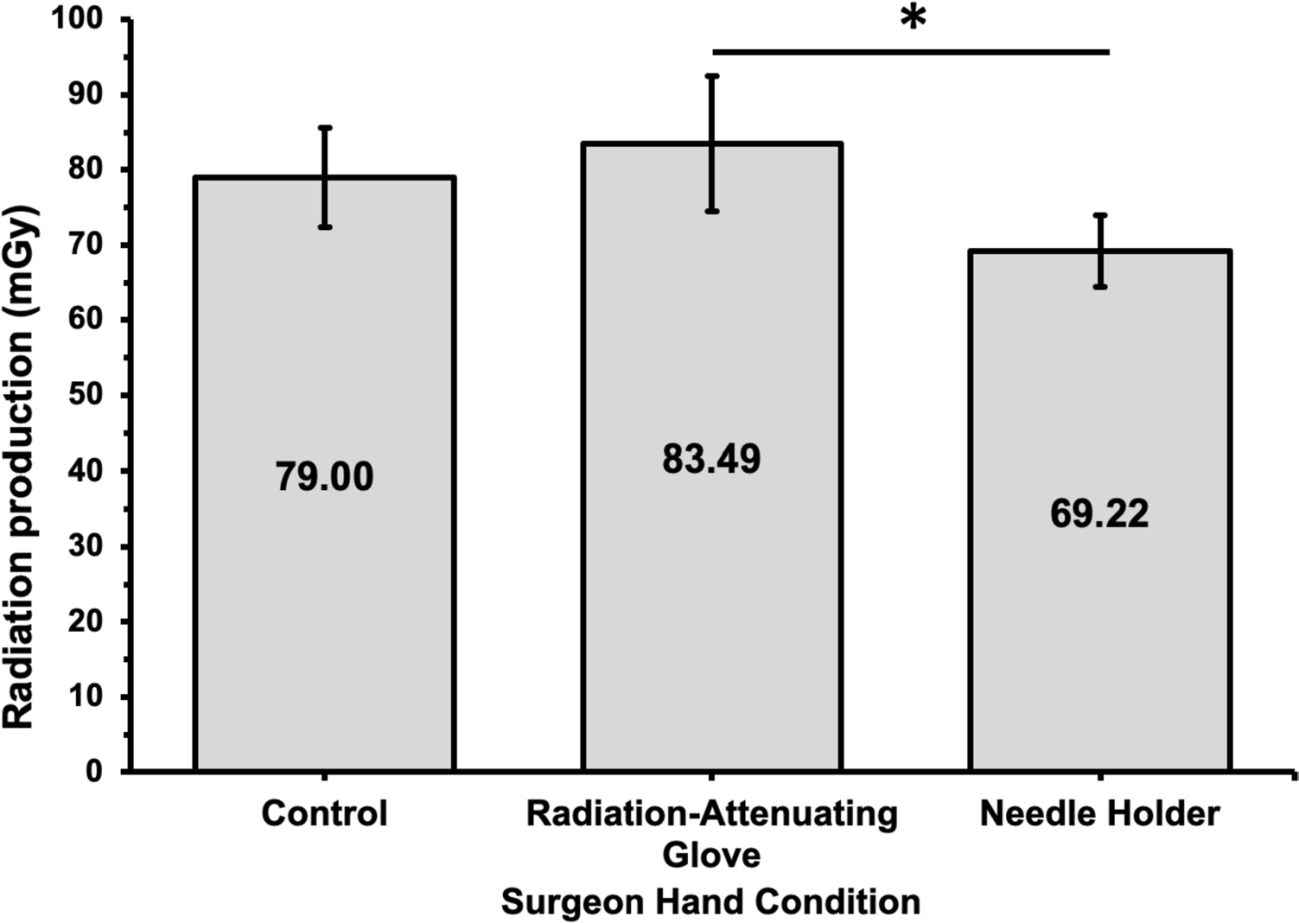
Mean radiation dose produced by fluoroscopy machine during the control, radiation-attenuating glove, and needle holder experimental arms for the surgeon hand model during simulated renal access for percutaneous nephrolithotomy

Using a radiation-attenuating glove resulted in a significant increase in the radiation generated by the fluoroscopy machine compared to using a needle holder (83.49 vs 69.22 mGy; *p* = 0.019; Fig. 5). Compared to the control (79.00 mGy), the radiation produced was higher with a radiation-attenuating glove and lower with a needle holder (*p* < 0.05 for all; Table 3). Although the kVp and mA were higher when using the radiation-attenuating glove and lower when using the needle holder compared to the control, there was no statistically significant difference (Table 3).

**Table 3:** Mean radiation dose, kVp, and mAs produced by the fluoroscopy machine between the control and radiation reduction techniques

	Control (*n* = 5)		Radiation-attenuating glove (*n* = 5)		Needle holder (*n* = 5)		*p* value
	Mean mGy (SD)	% Change vs control	Mean mGy (SD)	% Change vs control	Mean mGy (SD)	% Change vs control	
Radiation produced (mGy)	79.00 (6.57)	–	83.49 (9.0)	5.68	69.24 (4.75)	− 12.37	0.026*
kVp	93.60 (2.88)	–	95.2 (2.9)	1.71	90.4 (2.2)	− 3.42	0.083
mAs	3.09 (0.11)	–	3.12 (0.12)	1.29	2.95 (0.12)	− 4.53	0.116

Bold indicates statsitically significant *p*-value

*kVp* peak kilovoltage, *mAs* milliampere-seconds, *n* number of patients, *mGy* milligray, *SD* standard deviation

*p < 0.05

## Discussion

The existing literature extensively covers the various impacts of ionizing radiation such as DNA damage, malignancy, cataracts, dermatologic changes, and delayed wound healing [2–6, 21]. Despite this research evidence, there remains a significant gap in our understanding of the full health effects of chronic low-level radiation exposure [7, 8]. One area of specific concern is the potential damage to the hands of surgeons due to prolonged exposure to significant doses of ionizing radiation during procedures involving fluoroscopic-guided imaging. Urologists, who frequently use fluoroscopy to perform essential procedures, are particularly at risk for repeated low-level radiation exposure. Consequently, it becomes imperative to acknowledge the potential harm associated with all levels of ionizing radiation and take every possible protective measure to reduce exposure to as low as reasonably achievable (ALARA) [22]. In addition to posing a significant risk of joint, vascular, and skin pathologies, any adverse effects on a surgeon’s hand can significantly impact their ability to provide effective care and perform surgical procedures safely. To address these concerns and minimize potential harm, the ICRP guidelines suggest not exceeding 20 mSv of average radiation exposure per year over a 5-year period and an annual limit of 50 mSv. Additionally, the ICRP advises maintaining an annual radiation dose limit of 500 mSv to the skin and extremities [19].

Our study aimed to investigate the efficacy of radiation-attenuating gloves and a novel needle-holder in reducing ionizing radiation exposure to the surgeon’s hands during urological procedures. Using a cadaver model, we evaluated different techniques to reduce radiation exposure during simulated percutaneous renal access and observed significant exposure reductions to the surgeon’s hand. The use of a radiation-attenuating glove resulted in a 37% reduction, while employing a needle holder led to a 65% reduction in the mean equivalent dose to the surgeon’s hand (Table 1). The 37% radiation reduction for the radiation-attenuating glove at 95.2 kVp is comparable to the manufacturer specifications of 33% reduction at 100 kVp [20]. The decreased radiation dose associated with the radiation-attenuating glove may be attributed to the material’s high attenuation coefficient which reduces penetrating radiation. Similar reductions in exposure have been reported in orthopedics, where radiation-attenuating gloves were found to decrease exposure by 61% in an anthropomorphic model [21]. However, it is worth noting that their study used a mini-C arm with lower kVp and mA settings, unlike the GE OEC 9900 system at AEC settings used in our research. On the other hand, the needle holder further reduced exposure, likely by enabling the surgeon to remove their hand from the direct radiation beam. Notably, there is currently a lack of studies investigating the radiation-reducing effects of a needle holder during percutaneous renal access.

Simulated PCNL access demonstrated a mean radiation dose of 3.92 mSv to the hand without protection. Recent studies have reported similar hand radiation exposure during PCNL of 0.36–4.36 mSv [9, 23]. This wide range of hand exposure could be attributed to variations in fluoroscopy use, settings, and surgeon experience. Using the ICRP guidelines, approximately 127 PCNLs may be performed annually using surgical gloves without exceeding the dose limit for extremities. This number increases to 201 with radiation-attenuating gloves and 364 with the needle holder.

Custom-made needle holders have been specifically designed for the conventional “bullseye” technique in percutaneous renal access [24, 25]. As demonstrated in our study, these needle holders can potentially reduce radiation exposure to both the surgeon's hand and patient when constructed from a low radiation density material. However, literature on specialized needle holders for PCNL access is sparse. In contrast, the practice of utilizing needle holders has been extensively studied and more commonly employed in fields that frequently rely on fluoroscopic-guided imaging, such as interventional radiology. Studies investigating the use of improvised metal and custom-made plastic needle holders during fluoroscopic-guided interventions demonstrated significantly reduced radiation exposure to the user’s hand [26, 27]. In the field of endourology, the utilization of specialized needle holders is not yet widespread, requiring further research to determine their efficacy and safety.

Considerable research has been dedicated to reducing radiation exposure during fluoroscopic procedures in the operating room [1]. Implementation of simple yet effective measures, such as appropriate shielding, can lead to a significant reduction of up to 70-fold in radiation exposure [1]. Similarly, operating the fluoroscopy machine at lower power settings is another effective strategy [1]. However, adoption of these practices is not universal. A survey among endourologists revealed that lead aprons were worn in 99.3% of cases, thyroid shields in 98.7%, and radiation-attenuating gloves in only 9.7% [28]. The underuse of radiation-attenuating gloves is most likely multifactorial in nature, potentially due to cost, unacquaintance, inconvenience, or believing further protection is unnecessary due to current occupational dose limit guidelines. For fluoroscopy settings, the AEC setting remains the most commonly used mode due to its ability to obtain optimal quality images [29]. However, low dose modes and pulsed fluoroscopy are sufficient for many procedures [1].

The radiation reduction techniques explored in this study had implications not only for the surgeon but also for the patient. While the use of a radiation-attenuating glove reduced radiation exposure to the surgeon's hand by 37%, it resulted in a 3% increase in dose to the patient's dorsal surface (Table 1). In contrast, utilizing the needle holder reduced exposure for both the surgeon's hand by 65% and the patient's dorsal surface by 14%. The use of radiation-attenuating gloves may offer protection for the surgeon, but at the cost of increasing the dose to the patient. This is likely due to the effect of introducing hyperdense objects, such as radiation-attenuating gloves, into the path of the fluoroscopy beam. This has been shown to increase the radiation produced by the machine when it is operating in the AEC setting [14]. On the other hand, the needle holder is made from a low-density material and has a slim contour, which may have allowed the fluoroscopy machine to generate a lower radiation dose. This is supported by the findings regarding radiation dose, kVp, and mAs generated by the fluoroscopy machine in our study, which were found to be higher for the radiation-attenuating glove compared to the needle holder (Table 3).

Limitations of our study include the use of a cadaver model for simulated percutaneous renal access which cannot entirely replicate all aspects of the working environment encountered in a live PCNL. Nonetheless, this approach provided a controlled testing environment that allowed for accurate comparisons of radiation reduction techniques without resultant undue radiation exposure to human subjects. An additional limitation of our study is that it was designed to compare three different methods of holding the needle during fluoroscopic-guided access and did not include a comparison of ultrasound-guided access. We also used the AEC setting and a preset fluoroscopy time for simulated renal access. While the AEC setting is the most commonly used fluoroscopy setting, low-dose settings and pulsed fluoroscopy are also used in practice [1, 29]. The preset fluoroscopy time of 5 min in our study will not be representative of all practices and institutions. Finally, it is important to note that this needle holder and radiation-attenuating glove were only tested in a prone PCNL model, where the surgeon’s hand receives direct radiation exposure. In triangulation and supine PCNL the surgeon’s hand is less likely to encounter direct radiation exposure, and subsequently, these were not tested in our model. Despite these limitations, to our knowledge, this is the first study to assess and compare hand radiation reduction techniques during percutaneous renal access for PCNL in a controlled cadaver model.

## Conclusion

Protective measures during percutaneous renal access for PCNL can effectively reduce radiation exposure to a surgeon’s hand. Radiation-attenuating gloves showed a significant reduction in hand exposure but increased patient dose, while needle holders resulted in decreased exposure for both the surgeon and the patient. However, further research is needed to determine the efficacy and safety of radiation-attenuating gloves and needle holders in clinical urologic practice. These findings emphasize the importance of implementing radiation reduction techniques to enhance occupational safety during PCNL access.
